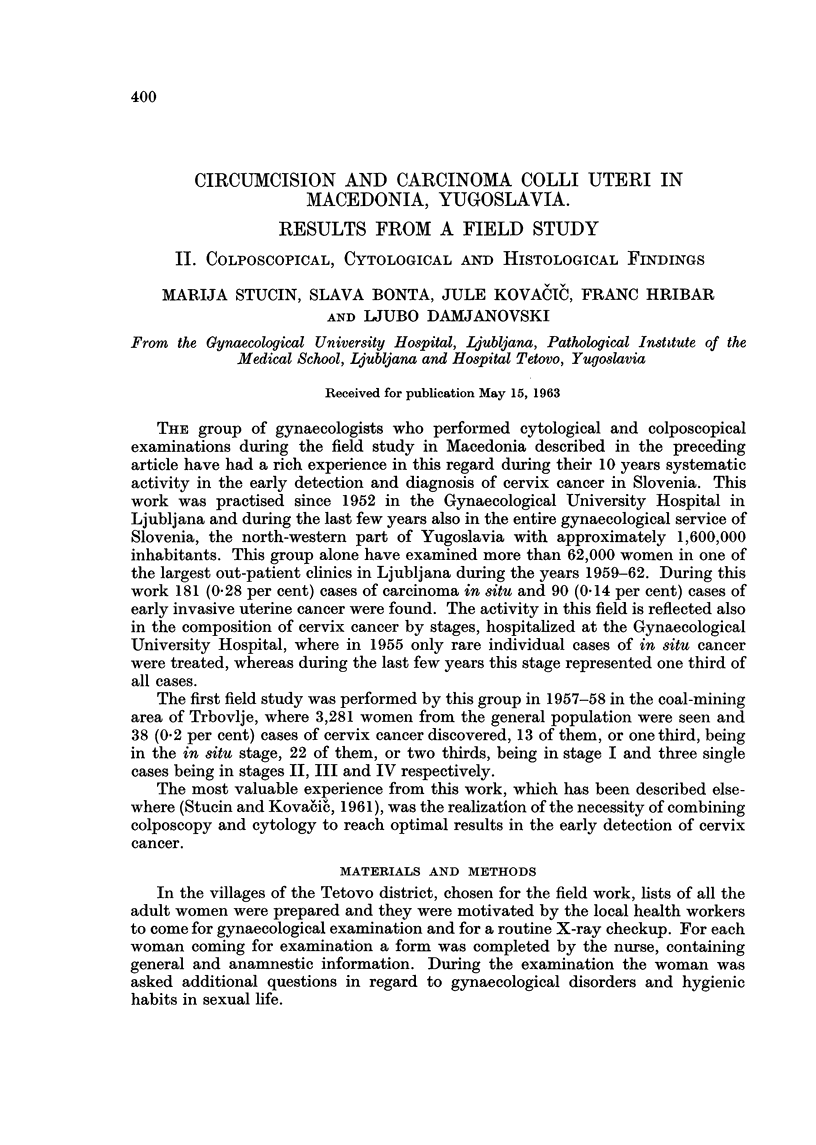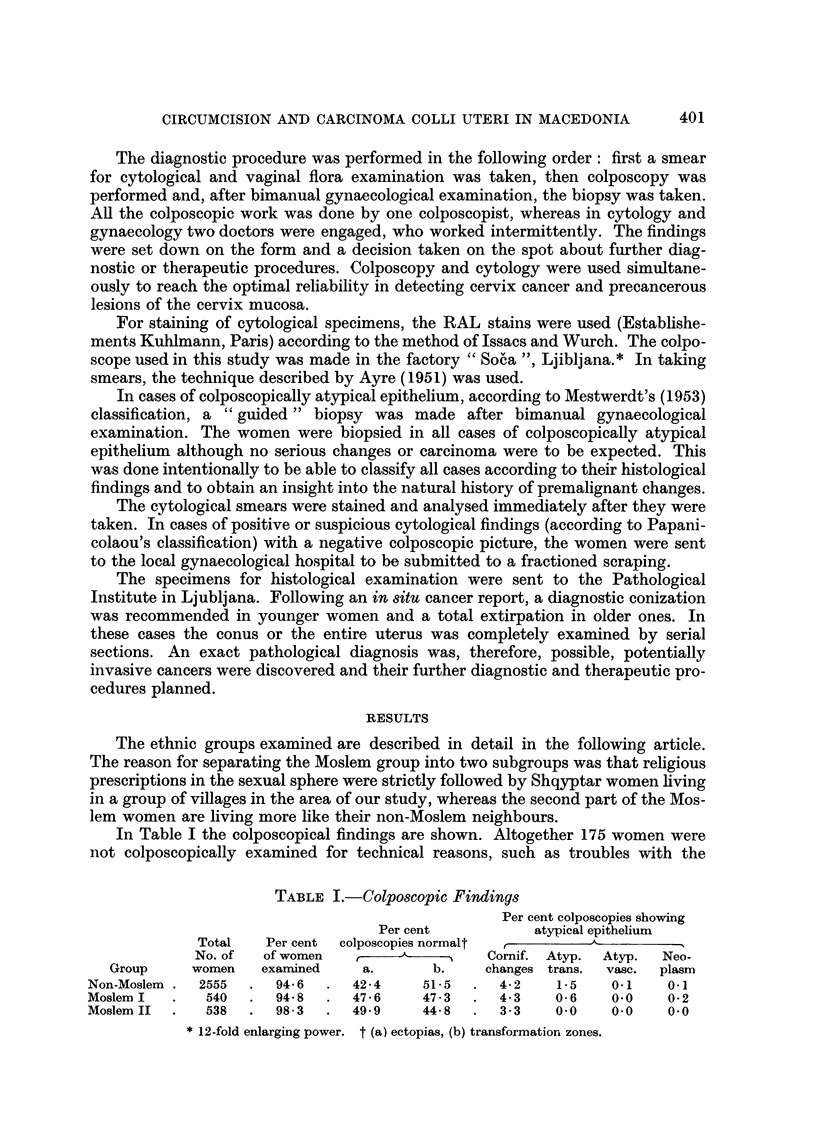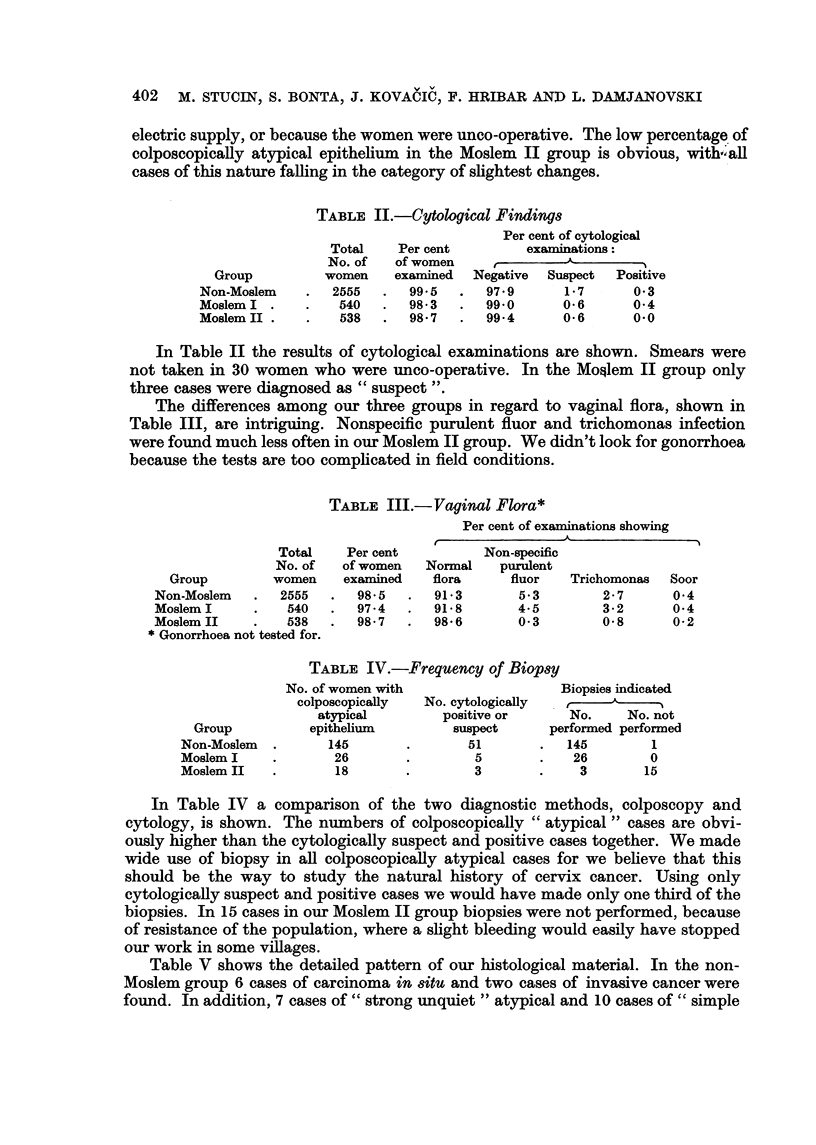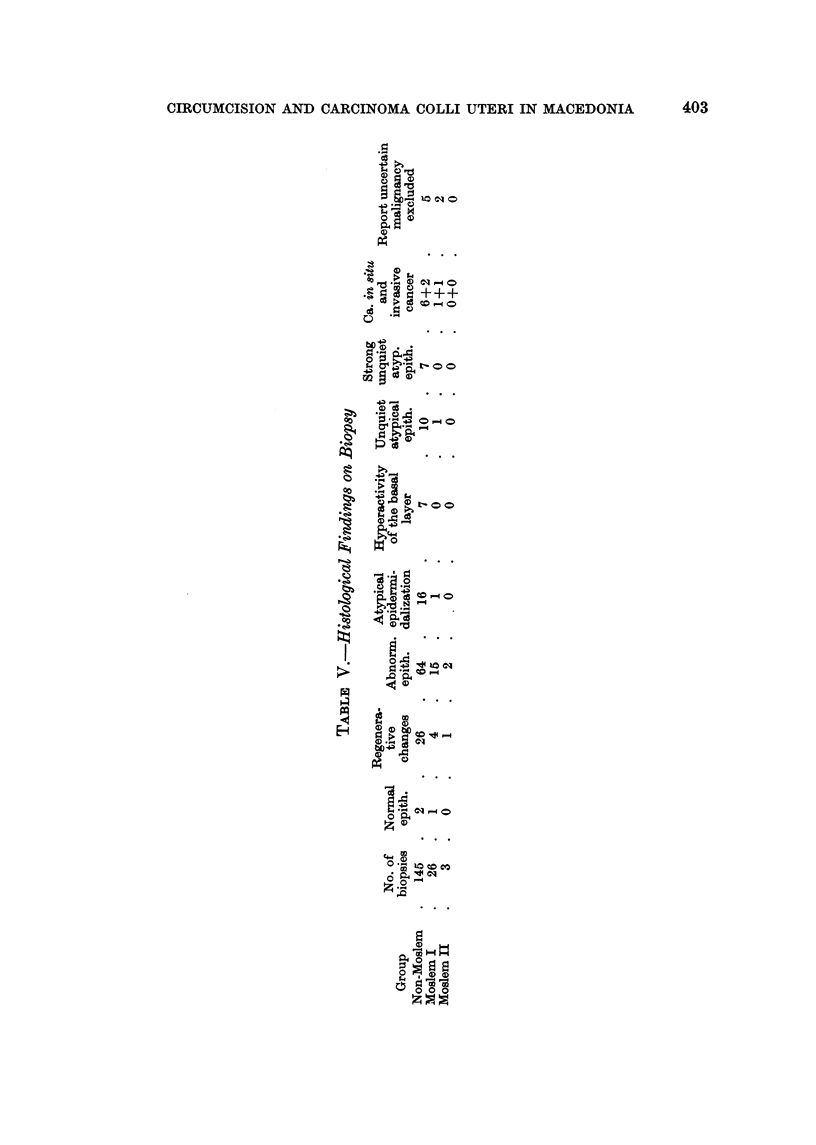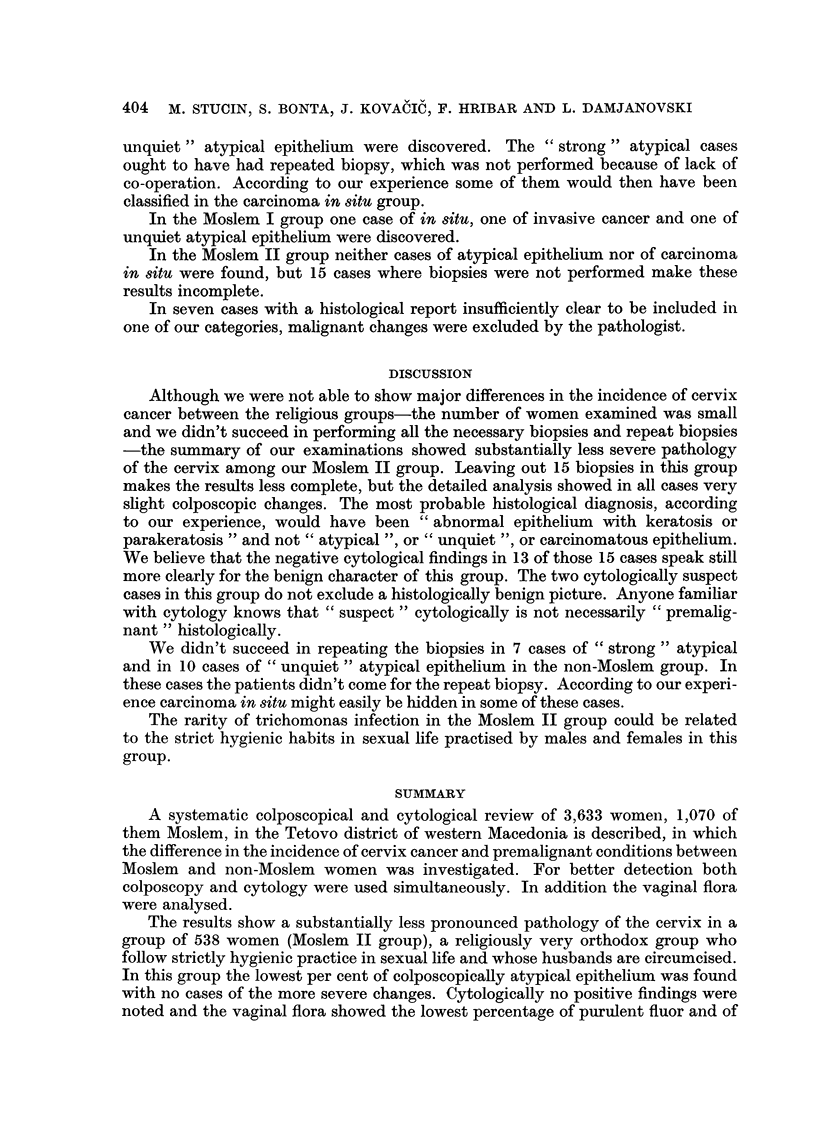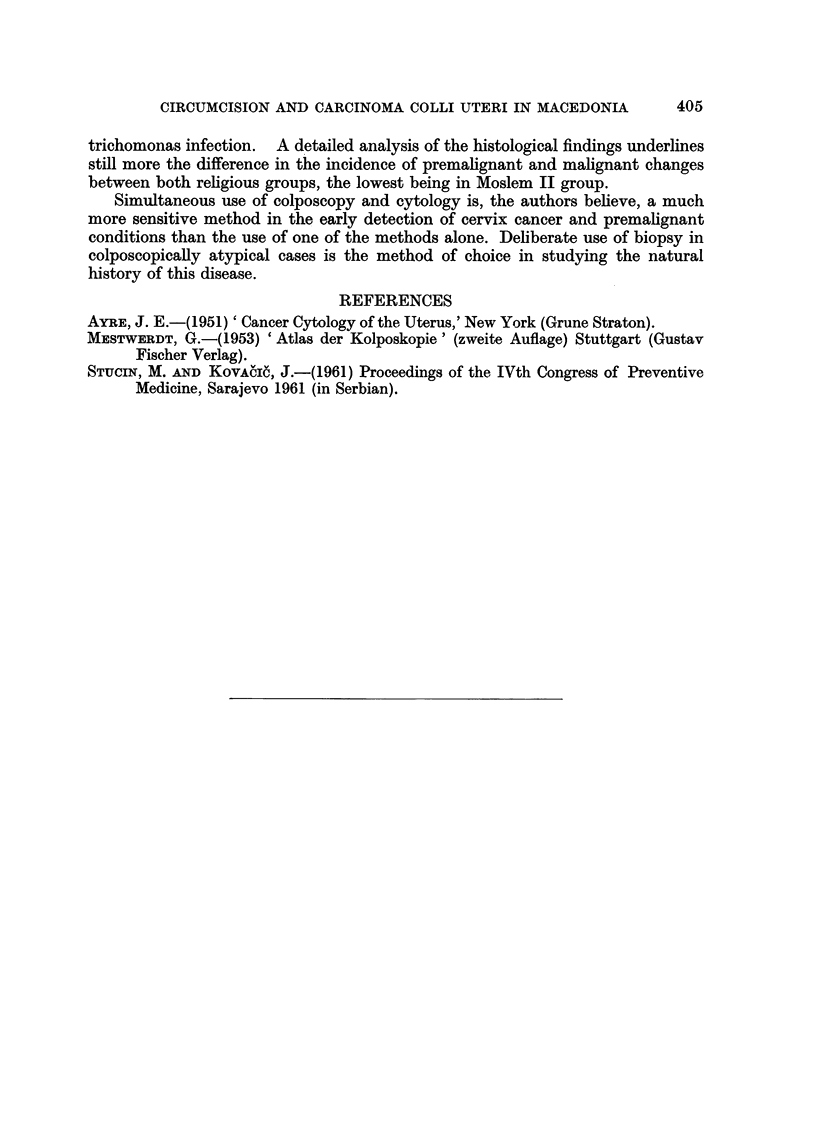# Circumcision and Carcinoma Colli Uteri in Macedonia, Yugoslavia. Results from a Field Study

**DOI:** 10.1038/bjc.1963.55

**Published:** 1963-09

**Authors:** Marija Stucin, Slava Bonta, Jule Kovačič, Franc Hribar, Ljubo Damjanovski


### CIRCUMCISION AND CARCINOMA COLLI UTERI IN MACEDONIA, YUGOSLAVIA. RESULTS FROM A FIELD STUDY

### II. Colposcopical, Cytological and Histological Findings

MARIJA STUCIN, SLAVA BONTA, JULE KOVAČIČ, FRANC HRIBAR AND LJUBO DAMJANOVSKI

*From the Gynaecological University Hospital, Ljubljana, Pathological Institute of the Medical School, Ljubljana and Hospital Tetovo, Yugoslavia*

Received for publication May 15, 1963

THE group of gynaecologists who performed cytological and colposcopical examinations during the field study in Macedonia described in the preceding article have had a rich experience in this regard during their 10 years systematic activity in the early detection and diagnosis of cervix cancer in Slovenia. This work was practised since 1952 in the Gynaecological University Hospital in Ljubljana and during the last few years also in the entire gynaecological service of Slovenia, the north-western part of Yugoslavia with approximately 1,600,000 inhabitants. This group alone have examined more than 62,000 women in one of the largest out-patient clinics in Ljubljana during the years 1959–62. During this work 181 (0·28 per cent) cases of carcinoma *in situ* and 90 (0·14 per cent) cases of early invasive uterine cancer were found. The activity in this field is reflected also in the composition of cervix cancer by stages, hospitalized at the Gynaecological University Hospital, where in 1955 only rare individual cases of *in situ* cancer were treated, whereas during the last few years this stage represented one third of all cases.

The first field study was performed by this group in 1957–58 in the coal-mining area of Trbovlje, where 3,281 women from the general population were seen and 38 (0·2 per cent) cases of cervix cancer discovered, 13 of them, or one third, being in the *in situ* stage, 22 of them, or two thirds, being in stage I and three single cases being in stages II, III and IV respectively.

The most valuable experience from this work, which has been described elsewhere (Stucin and Kovačič, 1961), was the realization of the necessity of combining colposcopy and cytology to reach optimal results in the early detection of cervix cancer.

### MATERIALS AND METHODS

In the villages of the Tetovo district, chosen for the field work, lists of all the adult women were prepared and they were motivated by the local health workers to come for gynaecological examination and for a routine X-ray checkup. For each woman coming for examination a form was completed by the nurse, containing general and anamnestic information. During the examination the woman was asked additional questions in regard to gynaecological disorders and hygienic habits in sexual life.

The diagnostic procedure was performed in the following order : first a smear for cytological and vaginal flora examination was taken, then colposcopy was performed and, after bimanual gynaecological examination, the biopsy was taken. All the colposcopic work was done by one colposcopist, whereas in cytology and gynaecology two doctors were engaged, who worked intermittently. The findings were set down on the form and a decision taken on the spot about further diagnostic or therapeutic procedures. Colposcopy and cytology were used simultaneously to reach the optimal reliability in detecting cervix cancer and precancerous lesions of the cervix mucosa.

For staining of cytological specimens, the RAL stains were used (Establishements Kuhlmann, Paris) according to the method of Issacs and Wurch. The colposcope used in this study was made in the factory " Soča ", Ljibljana.* In taking smears, the technique described by Ayre (1951) was used.

In cases of colposcopically atypical epithelium, according to Mestwerdt's (1953) classification, a " guided " biopsy was made after bimanual gynaecological examination. The women were biopsied in all cases of colposcopically atypical epithelium although no serious changes or carcinoma were to be expected. This was done intentionally to be able to classify all cases according to their histological findings and to obtain an insight into the natural history of premalignant changes.

The cytological smears were stained and analysed immediately after they were taken. In cases of positive or suspicious cytological findings (according to Papanicolaou's classification) with a negative colposcopic picture, the women were sent to the local gynaecological hospital to be submitted to a fractioned scraping.

The specimens for histological examination were sent to the Pathological Institute in Ljubljana. Following an *in situ* cancer report, a diagnostic conization was recommended in younger women and a total extirpation in older ones. In these cases the conus or the entire uterus was completely examined by serial sections. An exact pathological diagnosis was, therefore, possible, potentially invasive cancers were discovered and their further diagnostic and therapeutic procedures planned.

### RESULTS

The ethnic groups examined are described in detail in the following article. The reason for separating the Moslem group into two subgroups was that religious prescriptions in the sexual sphere were strictly followed by Shqyptar women living in a group of villages in the area of our study, whereas the second part of the Moslem women are living more like their non-Moslem neighbours.

In Table I the colposcopical findings are shown. Altogether 175 women were not colposcopically examined for technical reasons, such as troubles with the

TABLE I.—*Colposcopic Findings*

| Group | Total No. of women | Per cent of women examined | Per cent colposcopies normal† a. | b. | Per cent colposcopies showing atypical epithelium: Cornif. changes | Atyp. trans. | Atyp. vasc. | Neoplasm |
|---|---|---|---|---|---|---|---|---|
| Non-Moslem | 2555 | 94·6 | 42·4 | 51·5 | 4·2 | 1·5 | 0·1 | 0·1 |
| Moslem I | 540 | 94·8 | 47·6 | 47·3 | 4·3 | 0·6 | 0·0 | 0·2 |
| Moslem II | 538 | 98·3 | 49·9 | 44·8 | 3·3 | 0·0 | 0·0 | 0·0 |

* 12-fold enlarging power. † (a) ectopias, (b) transformation zones.

electric supply, or because the women were unco-operative. The low percentage of colposcopically atypical epithelium in the Moslem II group is obvious, with all cases of this nature falling in the category of slightest changes.

TABLE II.—*Cytological Findings*

| Group | Total No. of women | Per cent of women examined | Per cent of cytological examinations: Negative | Suspect | Positive |
|---|---|---|---|---|---|
| Non-Moslem | 2555 | 99·5 | 97·9 | 1·7 | 0·3 |
| Moslem I | 540 | 98·3 | 99·0 | 0·6 | 0·4 |
| Moslem II | 538 | 98·7 | 99·4 | 0·6 | 0·0 |

In Table II the results of cytological examinations are shown. Smears were not taken in 30 women who were unco-operative. In the Moslem II group only three cases were diagnosed as " suspect ".

The differences among our three groups in regard to vaginal flora, shown in Table III, are intriguing. Nonspecific purulent fluor and trichomonas infection were found much less often in our Moslem II group. We didn't look for gonorrhoea because the tests are too complicated in field conditions.

TABLE III.—*Vaginal Flora**

| Group | Total No. of women | Per cent of women examined | Per cent of examinations showing: Normal flora | Non-specific purulent fluor | Trichomonas | Soor |
|---|---|---|---|---|---|---|
| Non-Moslem | 2555 | 98·5 | 91·3 | 5·3 | 2·7 | 0·4 |
| Moslem I | 540 | 97·4 | 91·8 | 4·5 | 3·2 | 0·4 |
| Moslem II | 538 | 98·7 | 98·6 | 0·3 | 0·8 | 0·2 |

* Gonorrhoea not tested for.

TABLE IV.—*Frequency of Biopsy*

| Group | No. of women with colposcopically atypical epithelium | No. cytologically positive or suspect | Biopsies indicated: No. performed | No. not performed |
|---|---|---|---|---|
| Non-Moslem | 145 | 51 | 145 | 1 |
| Moslem I | 26 | 5 | 26 | 0 |
| Moslem II | 18 | 3 | 3 | 15 |

In Table IV a comparison of the two diagnostic methods, colposcopy and cytology, is shown. The numbers of colposcopically " atypical " cases are obviously higher than the cytologically suspect and positive cases together. We made wide use of biopsy in all colposcopically atypical cases for we believe that this should be the way to study the natural history of cervix cancer. Using only cytologically suspect and positive cases we would have made only one third of the biopsies. In 15 cases in our Moslem II group biopsies were not performed, because of resistance of the population, where a slight bleeding would easily have stopped our work in some villages.

Table V shows the detailed pattern of our histological material. In the non-Moslem group 6 cases of carcinoma *in situ* and two cases of invasive cancer were found. In addition, 7 cases of " strong unquiet " atypical and 10 cases of " simple

TABLE V.—*Histological Findings on Biopsy*

| Group | No. of biopsies | Normal epith. | Regenerative changes | Abnorm. epith. | Atypical epidermidalization | Hyperactivity of the basal layer | Unquiet atypical epith. | Strong unquiet atyp. epith. | Ca. *in situ* and invasive cancer | Report uncertain malignancy excluded |
|---|---|---|---|---|---|---|---|---|---|---|
| Non-Moslem | 145 | 2 | 26 | 64 | 16 | 7 | 10 | 7 | 6+2 | 5 |
| Moslem I | 26 | 1 | 4 | 15 | 1 | 0 | 1 | 0 | 1+1 | 2 |
| Moslem II | 3 | 0 | 1 | 2 | 0 | 0 | 0 | 0 | 0+0 | 0 |

unquiet " atypical epithelium were discovered. The " strong " atypical cases ought to have had repeated biopsy, which was not performed because of lack of co-operation. According to our experience some of them would then have been classified in the carcinoma *in situ* group.

In the Moslem I group one case of *in situ*, one of invasive cancer and one of unquiet atypical epithelium were discovered.

In the Moslem II group neither cases of atypical epithelium nor of carcinoma *in situ* were found, but 15 cases where biopsies were not performed make these results incomplete.

In seven cases with a histological report insufficiently clear to be included in one of our categories, malignant changes were excluded by the pathologist.

### DISCUSSION

Although we were not able to show major differences in the incidence of cervix cancer between the religious groups—the number of women examined was small and we didn't succeed in performing all the necessary biopsies and repeat biopsies—the summary of our examinations showed substantially less severe pathology of the cervix among our Moslem II group. Leaving out 15 biopsies in this group makes the results less complete, but the detailed analysis showed in all cases very slight colposcopic changes. The most probable histological diagnosis, according to our experience, would have been " abnormal epithelium with keratosis or parakeratosis " and not " atypical ", or " unquiet ", or carcinomatous epithelium. We believe that the negative cytological findings in 13 of those 15 cases speak still more clearly for the benign character of this group. The two cytologically suspect cases in this group do not exclude a histologically benign picture. Anyone familiar with cytology knows that " suspect " cytologically is not necessarily " premalignant " histologically.

We didn't succeed in repeating the biopsies in 7 cases of " strong " atypical and in 10 cases of " unquiet " atypical epithelium in the non-Moslem group. In these cases the patients didn't come for the repeat biopsy. According to our experience carcinoma *in situ* might easily be hidden in some of these cases.

The rarity of trichomonas infection in the Moslem II group could be related to the strict hygienic habits in sexual life practised by males and females in this group.

### SUMMARY

A systematic colposcopical and cytological review of 3,633 women, 1,070 of them Moslem, in the Tetovo district of western Macedonia is described, in which the difference in the incidence of cervix cancer and premalignant conditions between Moslem and non-Moslem women was investigated. For better detection both colposcopy and cytology were used simultaneously. In addition the vaginal flora were analysed.

The results show a substantially less pronounced pathology of the cervix in a group of 538 women (Moslem II group), a religiously very orthodox group who follow strictly hygienic practice in sexual life and whose husbands are circumcised. In this group the lowest per cent of colposcopically atypical epithelium was found with no cases of the more severe changes. Cytologically no positive findings were noted and the vaginal flora showed the lowest percentage of purulent fluor and of trichomonas infection. A detailed analysis of the histological findings underlines still more the difference in the incidence of premalignant and malignant changes between both religious groups, the lowest being in Moslem II group.

Simultaneous use of colposcopy and cytology is, the authors believe, a much more sensitive method in the early detection of cervix cancer and premalignant conditions than the use of one of the methods alone. Deliberate use of biopsy in colposcopically atypical cases is the method of choice in studying the natural history of this disease.

### REFERENCES

AYRE, J. E.—(1951) ‘ Cancer Cytology of the Uterus,’ New York (Grune Straton).

MESTWERDT, G.—(1953) ‘ Atlas der Kolposkopie ’ (zweite Auflage) Stuttgart (Gustav Fischer Verlag).

STUCIN, M. AND KOVAČIČ, J.—(1961) Proceedings of the IVth Congress of Preventive Medicine, Sarajevo 1961 (in Serbian).